# Radiographic evidence of thymic involution in hepatocellular carcinoma: a propensity score-matched study in viral cirrhosis

**DOI:** 10.3389/fimmu.2026.1937686

**Published:** 2026-08-28

**Authors:** Jin Li, Yawhan Lakang, Jin-Xiang Zuo, Hai Li, Wan-Ling Luo, Xing-Ming Chen, Zi-Sheng Yang, Hao-Bo Xu, Jin-Yi Zhou, Yu-Bo Liang, Qing-Bo Wang, Yu-Kai Li, Wei Wang, Chen Yang, Li Guo, Xin-Xiang Zhao, Yang Ke

**Affiliations:** 1 Yunnan Key Laboratory of Stem Cell and Regenerative Medicine, Department of Hepatobiliary Surgery, the Second Affiliated Hospital of Kunming Medical University, Kunming, Yunnan, China; 2 Department of Reproductive Medicine, the Second Affiliated Hospital of Kunming Medical University, Kunming, Yunnan, China; 3 Department of Liver Transplantation, the First People’s Hospital of Yunnan, Kunming, Yunnan, China; 4 Department of International Healthcare, the First Affiliated Hospital of Kunming Medical University, Kunming, Yunnan, China; 5 Department of Radiology, the Second Affiliated Hospital of Kunming Medical University, Kunming, Yunnan, China; 6 Department of Surgical Education and Research, the Second Affiliated Hospital of Kunming Medical University, Kunming, Yunnan, China; 7 Yunnan Yunke Bio-Technology Institution, Kunming, Yunnan, China

**Keywords:** cirrhosis, immunosenescence, liver cancer, thymic involution, tumorigenesis

## Abstract

**Purpose:**

Adult thymic involution has been implicated in impaired antitumor immunity, but whether hepatocellular carcinoma (HCC) is associated with structural thymic involution in humans remains unclear. This study evaluated parameters of thymic CT imaging in compensated liver viral cirrhotic patients with or without concomitant HCC.

**Patients and methods:**

Patients with liver cirrhosis, with or without concomitant HCC, diagnosed between January 2016 and December 2022 were retrospectively screened. To minimize confounding from liver disease background, all patients were restricted to Child-Pugh class A cirrhosis of viral etiology. CT-derived thymic parameters, including anteroposterior and transverse diameters, left/right and overall bilateral lobe length and thickness, and CT attenuation, were measured using the Thymic Measurement and Analysis Program (ThyMAP). Propensity score matching (PSM) was performed to balance age, sex, hepatitis virus infection status, and Child-Pugh score. Sex-stratified and Barcelona Clinic Liver Cancer (BCLC) stage-stratified analyses were also conducted.

**Results:**

A total of 121 patients with cirrhosis without HCC and 223 patients with cirrhosis-related HCC were included. Before PSM, patients with HCC had significantly smaller anteroposterior diameter, right lobe length, overall bilateral lobe length, left lobe thickness, right lobe thickness, and overall bilateral lobe thickness, compared with those without HCC. After PSM, 115 matched pairs were generated, and all measured thymic dimensions were significantly reduced in the HCC group. CT attenuation did not differ significantly before or after PSM. In sex-stratified analyses, significant reductions in several thymic dimensions were observed among male patients, whereas no significant differences were detected among female patients. Among patients with HCC, thymic parameters did not differ significantly between BCLC stages 0–A and B–C.

**Conclusion:**

In patients with compensated viral cirrhosis, HCC occurrence is associated with reduced CT-derived thymic dimensions, but not CT attenuation. This association is more pronounced in male patients and appears related to HCC presence rather than BCLC stage progression.

## Introduction

Hepatocellular carcinoma (HCC) is a highly prevalent and lethal malignancy (1–4). Approximately 80% of patients with HCC are male, and most HCC cases have a history of chronic liver disease and/or cirrhosis (5–8). HCC is regarded as a prototypical inflammation-driven and immunoreactive malignancy, and HCC initiation and progression are regulated by immune evasion (9–13). In recent years, immunotherapy has emerged as an important treatment modality for HCC (14–20). Because both endogenous antitumor immune control and the efficacy of immunotherapy in HCC ultimately depend on the development and function of competent T cells (21–25), the role of the thymus—the primary organ responsible for T-cell development and maturation—in HCC development and progression warrants closer attention (26, 27).

The thymus, however, does not retain a stable structure throughout life. Under physiological conditions, its weight and volume peak around puberty. Thereafter, the thymus undergoes progressive involution accompanied by adipose replacement, and by middle age the parenchyma is predominantly replaced by fat (28, 29). A growing body of evidence indicates that the extent of this structural atrophy is closely associated with increased cancer risk and poorer outcomes across multiple cancer types. Lower CT-based thymic health, as a radiographic surrogate of thymic involution, has been associated with increased incidence and high mortality of lung cancer, as well as poorer outcomes of multiple types of cancers following immune checkpoint inhibitor therapy (30, 31). As a more extreme example of thymic loss, adult thymectomy has been associated with increased risk of cancer incidence and mortality (32, 33). Taken together, these findings suggest that adult thymic integrity is not merely an anatomical remnant but a clinically relevant host factor in antitumor immune surveillance and immunotherapy responsiveness.

The relevance of thymic integrity to antitumor immunity is particularly pertinent to HCC, given its immune-driven pathogenesis (34, 35). Notably, the relationship between the thymus and HCC development may even be bidirectional: a mouse study has shown that HCC itself can promote thymic involution (36). Evidently, in a mouse model of subcutaneously axillary H22 HCC, tumor-bearing mice exhibited significant reductions in thymus weight and thymic index compared with tumor-free control mice. These alterations were accompanied by disruption of the thymic epithelial network and increased extracellular matrix deposition, suggesting that H22 solid tumors may enhance structural thymic atrophy (36). However, whether HCC occurrence is associated with structural thymic involution in humans remains unknown.

Here, we conducted a retrospective study to investigate whether HCC occurrence is associated with thymic involution, using CT-derived thymic parameters—including dimensions and CT attenuation—in patients with liver cirrhosis. To minimize potential confounding effects from the severity and etiology of underlying liver diseases, all enrolled patients were restricted to Child-Pugh class A cirrhosis of viral origin, and their age, sex, hepatitis virus infection status, and Child-Pugh score were subjected to propensity score matching (PSM). This design allowed us to isolate the association between HCC occurrence and thymic involution under a unified hepatic disease background.

## Materials and methods

This retrospective study was approved by the Ethics Committee of the Second Affiliated Hospital of Kunming Medical University (approval no. Shen-PJ-KY-2026-75) and was conducted in accordance with the Declaration of Helsinki (37–41). The requirement for informed consent was waived by the Ethics Committee owing to the retrospective design of the study.

### Patients

Patients in the cirrhosis-without-HCC group were selected according to the following inclusion and exclusion criteria.

The inclusion criteria were: (1) patients aged between 18 and 75 years, regardless of sex; (2) patients with liver cirrhosis were initially diagnosed at the Department of Gastroenterology, Second Affiliated Hospital of Kunming Medical University, between January 2016 and December 2022. The diagnosis of cirrhosis was based on the presence of at least one of the following features: histologically confirmed METAVIR stage F4 fibrosis, liver stiffness measurement >15 kPa by FibroScan, platelet count <100 × 10^9^/L, or the presence of gastroesophageal varices or splenomegaly (42–44); (3) patients with compensated liver cirrhosis (Child-Pugh class A, 5–6 points) without ascites, hepatic encephalopathy, esophagogastric variceal bleeding, hepatorenal syndrome, or sepsis; (4) patients whose liver cirrhosis was attributable to hepatitis B virus (HBV) and/or hepatitis C virus (HCV) infection; and (5) patients who underwent chest CT examination (with or without contrast enhancement) at the time of initial cirrhosis diagnosis.

The exclusion criteria were: (1) patients with concomitant thymic or anterior mediastinal diseases, including benign or malignant thymic tumors; (2) patients with concomitant immune-related disorders, such as autoimmune liver diseases (autoimmune hepatitis, primary biliary cholangitis, and primary sclerosing cholangitis) (45, 46), HIV infection, systemic lupus erythematosus, or myasthenia gravis; (3) patients with liver disease of non-viral etiology, such as alcoholic liver disease, non-alcoholic fatty liver disease (NAFLD; now termed metabolic dysfunction-associated steatotic liver disease [MASLD]), or schistosomiasis; (4) patients who received continuous immunosuppressant or glucocorticoid therapy for more than two weeks within three months before the chest CT examination; and (5) patients with a concomitant malignant tumor or another life-threatening disease.

Patients in the cirrhosis-with-HCC group were selected according to the following inclusion and exclusion criteria.

The inclusion criteria were: (1) patients aged between 18 and 75 years, regardless of sex; (2) patients initially diagnosed with HCC at Barcelona Clinic Liver Cancer (BCLC) stages 0-C who underwent initial radical resection at the Department of Hepatobiliary Surgery, Second Affiliated Hospital of Kunming Medical University, between January 2016 and December 2022; (3) patients with compensated liver cirrhosis (Child-Pugh class A, 5–6 points) without ascites, hepatic encephalopathy, esophagogastric variceal bleeding, hepatorenal syndrome, or sepsis; (4) patients whose liver cirrhosis was attributable to HBV and/or HCV infection; (5) patients who underwent preoperative chest CT examination (with or without contrast enhancement).

The exclusion criteria were: (1) patients who received any neoadjuvant treatment for HCC before the preoperative chest CT examination, such as transcatheter arterial chemoembolization, transcatheter arterial embolization, transcatheter arterial infusion, hepatic arterial infusion chemotherapy, or targeted therapy and/or immunotherapy; (2) patients with concomitant thymic or anterior mediastinal diseases, including benign or malignant thymic tumors; (3) patients with concomitant immune-related disorders, such as autoimmune liver diseases (autoimmune hepatitis, primary biliary cholangitis, and primary sclerosing cholangitis) (45, 46), HIV infection, systemic lupus erythematosus, or myasthenia gravis; (4) patients with liver disease of non-viral etiology, such as alcoholic liver disease, NAFLD (now termed MASLD), or schistosomiasis; (5) patients who received continuous immunosuppressant or glucocorticoid therapy for more than two weeks within three months before the chest CT examination; (6) patients with a concomitant malignant tumor other than HCC, or another life-threatening disease.

### Data collection and assessment of thymic CT parameters

Demographic and clinical data were retrieved from the electronic medical record system of the Second Affiliated Hospital of Kunming Medical University. Demographic parameters included sex (47, 48) and age, which was categorized as < 60 years or ≥ 60 years (49–51). Clinical data comprised hepatitis virus infection status, the Child-Pugh score, and, for patients with HCC, the BCLC stage (52–55). All were recorded at enrollment. HBV infection was defined by positivity for hepatitis B surface antigen (HBsAg) and HCV infection by positivity for anti-HCV antibody; patients positive for both were classified as having HBV-HCV co-infection (56, 57). Within Child-Pugh class A, their Child-Pugh scores were further categorized as 5 or 6 (58, 59).

**Figure 1:**
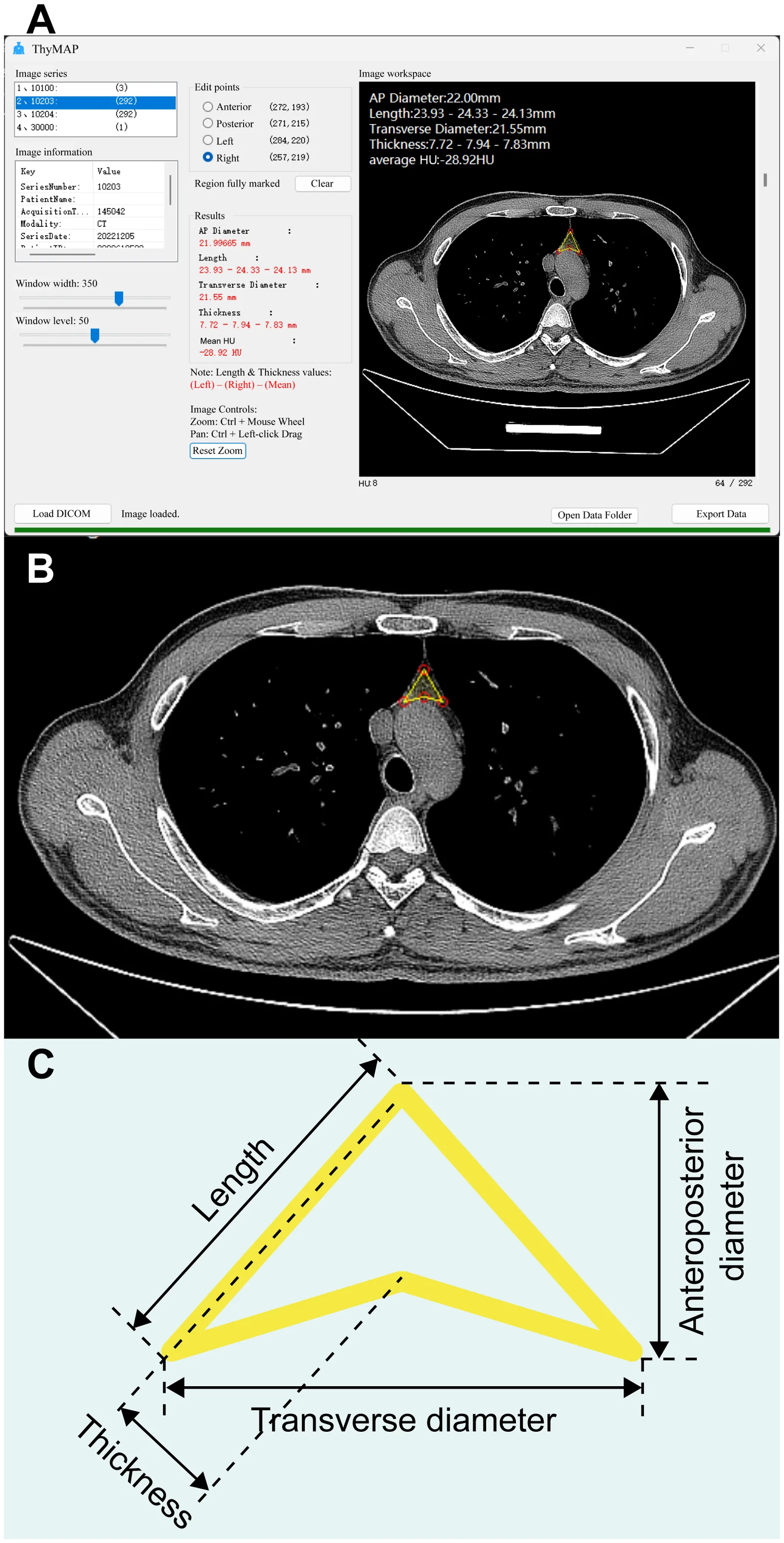
Measurement of thymic parameters using the Thymic Measurement and Analysis Program (ThyMAP). **(A)** The ThyMAP interface, which displays the loaded DICOM series, image information, window settings, and the computed results (anteroposterior [AP] diameter, length, transverse diameter, thickness, and mean HU). **(B)** Representative axial CT image at the level of the maximum thymic AP diameter, which was selected for measurement. An arrowhead-shaped region of interest (ROI) was delineated using four manually placed points corresponding to the anterior, posterior, left, and right margins of the thymus (shown in red). **(C)** Schematic illustrating the measurement of the AP diameter, transverse diameter, length, and thickness within the arrowhead-shaped ROI; length and thickness were measured separately for the left and right lobes.

We developed computer-assisted thymic measurement software in C# on the .NET Framework 4.7, using WinForms and GDI+. The software was named ThyMAP (Thymic Measurement and Analysis Program; Software Copyright Registration No. 2026SR0215070; **Figure 1A**). The measurement protocol implemented in ThyMAP was adapted from previously established CT-based methods for assessing thymic morphology (60), and the software-derived measurements were validated for consistency against manual measurements.

Chest CT Digital Imaging and Communications in Medicine (DICOM) data for eligible patients were retrieved from the Picture Archiving and Communication System (PACS) of the Second Affiliated Hospital of Kunming Medical University and imported into ThyMAP. All assessments were performed on axial CT images in the mediastinal window (window width, 350 HU; window level, 50 HU). Following the method of Araki et al. (60), the slice showing the maximum anteroposterior diameter of the thymus was selected for all measurements. Because the thymus is most commonly arrowhead-shaped (60–62), an arrowhead-shaped region of interest (ROI) was manually delineated with four points to cover the largest cross-sectional area of the thymus while minimizing the inclusion of adjacent mediastinal adipose tissue (**Figure 1B**). Using ThyMAP, thymic dimensions were measured in a blinded manner, including the anteroposterior and transverse diameters; left and right lobe thicknesses; and left and right lobe lengths (**Figure 1C**). The mean CT attenuation within the thymic ROI was also calculated for each patient (63–65). CT attenuation measurements were based exclusively on non-contrast CT images.

### Statistical analysis

Data were analyzed using IBM SPSS Statistics, version 26.0 (IBM Corp., Armonk, NY, USA).

No missing data were identified for any of the variables included in the statistical analyses; therefore, no imputation or other procedures for handling missing data were required. To reduce selection bias and residual confounding, demographic and clinical data, including age, sex, hepatitis virus infection status, and Child-Pugh score, were subjected to PSM using the Propensity Score Matching extension for SPSS (version 3.0.4), which is based on the R package MatchIt (66, 67). A logistic regression model was used to estimate propensity scores. PSM was conducted using 1:1 nearest-neighbor matching with a caliper of 0.01 standard deviations (SDs) of the logit of the propensity score. Between-group balance before and after matching was assessed using the standardized mean difference (SMD), with an SMD below 0.100 considered indicative of satisfactory balance. Kernel density plots were generated to visually compare the distributions of propensity scores before and after matching.

Normally distributed continuous variables were presented as mean ± SD, whereas skewed continuous data were presented as median and interquartile range (IQR). Categorical variables were expressed as counts and percentages. For descriptive analysis, left and right lobe lengths were summarized jointly and reported as overall bilateral lobe length; similarly, left and right lobe thicknesses were summarized jointly and reported as overall bilateral lobe thickness. Differences between groups in the unmatched cohort were analyzed using an independent-samples t-test or the Mann-Whitney U test, as appropriate. Categorical variables were compared using the chi-squared test or Fisher’s exact test, as appropriate. In the propensity score-matched cohort, the paired comparisons were performed using the paired t-test or Wilcoxon signed-rank test for continuous variables, and McNemar’s test or McNemar–Bowker test for categorical variables, as appropriate (68–70). A two-sided P-value of < 0.05 was considered statistically significant.

## Results

### Patient characteristics

**Figure 2:**
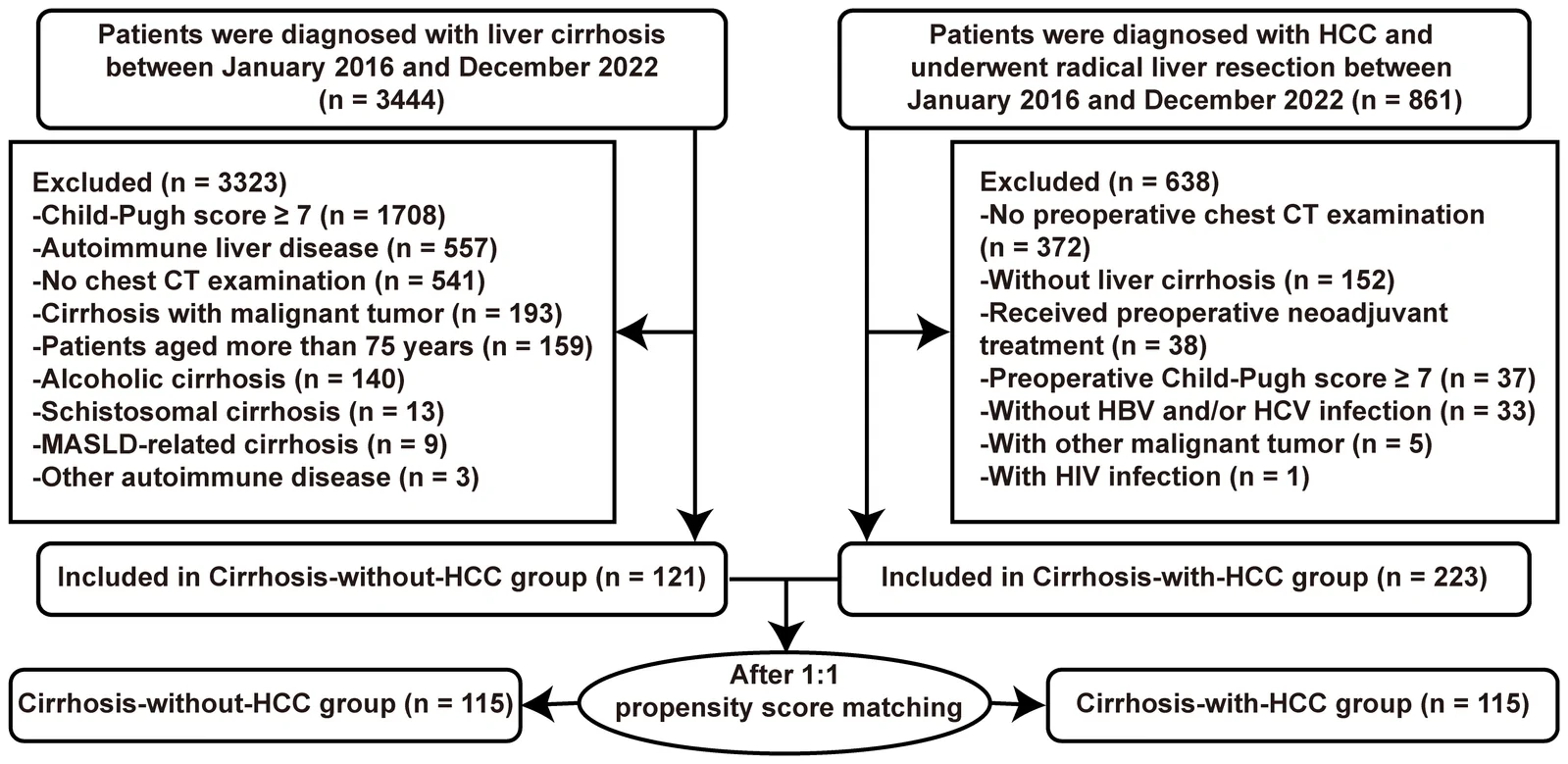
The flowchart of patient selection for the study. CT, computed tomography; HBV, hepatitis B virus; HCC, hepatocellular carcinoma; HCV, hepatitis C virus; HIV, human immunodeficiency virus; MASLD, metabolic dysfunction-associated steatotic liver disease.

A total of 3,444 patients were diagnosed with liver cirrhosis at the Department of Gastroenterology of the Second Affiliated Hospital of Kunming Medical University, between January 2016 and December 2022, and were retrospectively screened for eligibility (**Figure 2**). Of them, 3,323 patients were excluded for the following reasons: Child-Pugh score ≥ 7 (n = 1,708), autoimmune liver disease (n = 557), absence of chest CT at initial diagnosis (n = 541), concomitant malignancy (n = 193), age > 75 years (n = 159), alcoholic cirrhosis (n = 140), schistosomiasis-induced cirrhosis (n = 13), MASLD-related cirrhosis (n = 9), and other non-hepatic autoimmune diseases (n = 3). The remaining 121 patients were included in the cirrhosis-without-HCC group.

A total of 861 patients were diagnosed with HCC and consecutively underwent radical liver resection at the Second Affiliated Hospital of Kunming Medical University between January 2016 and December 2022. Those patients were retrospectively screened for eligibility (**Figure 2**). Of them, 638 patients were excluded for the following reasons: absence of preoperative chest CT (n = 372), HCC without cirrhosis (n = 152), neoadjuvant treatment before the chest CT examination (n = 38), Child-Pugh score ≥ 7 (n = 37), absence of evidence for HBV and/or HCV infection (n = 33), concurrent malignancies other than HCC (n = 5), and HIV infection (n = 1). The remaining 223 patients were included in the cirrhosis-with-HCC group.

The demographic and clinical characteristics of these two groups of patients are summarized in **Table 1**. Male patients accounted for 71.1% (86/121) of the cirrhosis-without-HCC group and 85.7% (191/223) of the cirrhosis-with-HCC group (*P* = 0.001). The proportion of patients aged ≥60 years was comparable between these two groups (20.7% vs. 22.0%, *P* = 0.777). The distribution of hepatitis virus infection also did not differ significantly between groups (*P* = 0.833), with HBV-only infection predominating in both the cirrhosis-without-HCC group (85.1%) and the cirrhosis-with-HCC group (84.3%), followed by HCV-only infection (14.9% vs. 14.8%) and HBV-HCV co-infection (0% vs. 0.9%). The proportion with a Child-Pugh score of 5 was likewise similar (76.0% vs. 81.2%, *P* = 0.261). Among the 223 patients in the cirrhosis-with-HCC group, 116 (52.0%) were classified as BCLC stage 0–A, 51 (22.9%) as BCLC stage B, and 56 (25.1%) as BCLC stage C.

**Table 1:** Baseline clinical characteristics and thymic imaging parameters in cirrhotic patients with and without HCC.

Variable	Cirrhosis-without-HCC group (n=121)	Cirrhosis-with-HCC group (n=223)	*P*
Sex			0.001
Male	86 (71.1%)	191 (85.7%)	
Female	35 (28.9%)	32 (14.3%)	
Age (year)			0.777
≥60	25 (20.7%)	49 (22.0%)	
<60	96 (79.3%)	174 (78.0%)	
Hepatitis virus infection			0.833
HBV-only	103 (85.1%)	188 (84.3%)	
HCV-only	18 (14.9%)	33 (14.8%)	
HBV + HCV	0 (0.0%)	2 (0.9%)	
Child-Pugh score			0.261
5	92 (76.0%)	181 (81.2%)	
6	29 (24.0%)	42 (18.8%)	
BCLC stage			-
0-A	–	116 (52.0%)	
B	–	51 (22.9%)	
C	–	56 (25.1%)	
AP diameter (mm)	13.4 (10.2, 16.9)	12.1 (9.3, 15.3)	0.017
Transverse diameter (mm)	22.5 (16.9, 28.3)	20.7 (14.8, 28.2)	0.173
Left lobe length (mm)	17.4 (14.2, 21.2)	16.0 (12.6, 20.7)	0.055
Right lobe length (mm)	17.5 (14.1, 21.3)	16.3 (12.9, 19.9)	0.024
Overall bilateral lobe length (mm)	17.5 (14.2, 21.1)	16.1 (12.8, 20.2)	0.003
Left lobe thickness (mm)	6.7 (5.6, 8.5)	6.2 (5.0, 7.3)	<0.001
Right lobe thickness (mm)	6.9 (5.5, 8.4)	6.4 (5.1, 7.6)	0.011
Overall bilateral lobe thickness (mm)	6.8 (5.6, 8.5)	6.2 (5.1, 7.5)	<0.001
CT attenuation (HU)	-57.3 (-80.2, -26.7)	-58.7 (-81.8, -38.0)	0.268

AP, anteroposterior; BCLC, Barcelona Clinic Liver Cancer; CT, computed tomography; HBV, hepatitis B virus; HCC, hepatocellular carcinoma; HCV, hepatitis C virus.

### Thymic imaging parameters in cirrhotic patients with or without HCC

ThyMAP-derived measurements showed excellent agreement with manual measurements, with intraclass correlation coefficients ranging from 0.90 to 0.92. The parameters of thymic CT imaging of these two groups of patients are presented in **Table 1**. Compared with the cirrhosis-without-HCC group, the cirrhosis-with-HCC group exhibited significantly reduced anteroposterior diameter (*P* = 0.017), right lobe length (*P* = 0.024), overall bilateral lobe length (*P* = 0.003), left lobe thickness (*P* < 0.001), right lobe thickness (*P* = 0.011), and overall bilateral lobe thickness (*P* < 0.001). However, there was no significant difference in the values of thymic transverse diameter (*P* = 0.173), left lobe length (*P* = 0.055), or CT attenuation (*P* = 0.268) between these two groups of patients.

### Thymic imaging parameters in male cirrhotic patients with and without HCC

Given the significant imbalance in the distribution of sex between groups, a sex-stratified analysis was performed: thymic imaging parameters were compared between cirrhotic patients with and without HCC separately within the male and female cohorts.

Within the male cohort, no significant differences were observed between groups in the distribution of age (*P* = 0.214), hepatitis virus infection status (*P* = 0.932), or Child-Pugh score (*P* = 0.398) (**Table 2**). Compared with the cirrhosis-without-HCC group, male patients in the cirrhosis-with-HCC group exhibited significantly lower anteroposterior diameter (*P* = 0.033), transverse diameter (*P* = 0.031), right lobe length (*P* < 0.001), overall bilateral lobe length (*P* < 0.001), left lobe thickness (*P* < 0.001), right lobe thickness (*P* < 0.001), and overall bilateral lobe thickness (*P* < 0.001). No significant difference was found in left lobe length (*P* = 0.057) and CT attenuation (*P* = 0.188).

**Table 2:** Baseline clinical characteristics and thymic imaging parameters in male cirrhotic patients with and without HCC.

Variable	Cirrhosis-without-HCC group (n=86)	Cirrhosis-with-HCC group (n=191)	*P*
Age (year)			0.214
≥60	11 (12.8%)	36 (18.8%)	
<60	75 (87.2%)	155 (81.2%)	
Hepatitis virus infection			0.932
HBV-only	74 (86.0%)	160 (83.8%)	
HCV-only	12 (14.0%)	29 (15.2%)	
HBV + HCV	0 (0.0%)	2 (1.0%)	
Child-Pugh score			0.398
5	66 (76.7%)	155 (81.2%)	
6	20 (23.3%)	36 (18.8%)	
BCLC stage			-
0-A	–	96 (50.3%)	
B	–	45 (23.5%)	
C	–	50 (26.2%)	
AP diameter (mm)	13.8 (10.2, 17.0)	12.3 (9.4, 15.4)	0.033
Transverse diameter (mm)	23.2 (17.0, 28.1)	20.4 (14.7, 27.0)	0.031
Left lobe length (mm)	17.8 (14.2, 21.2)	16.1 (12.6, 20.2)	0.057
Right lobe length (mm)	18.3 (15.3, 22.7)	16.3 (12.9, 19.8)	<0.001
Overall bilateral lobe length (mm)	17.9 (14.8, 21.8)	16.2 (12.8, 20.1)	<0.001
Left lobe thickness (mm)	7.0 (5.9, 9.1)	6.2 (5.0, 7.4)	<0.001
Right lobe thickness (mm)	7.3 ± 2.2	6.5 ± 1.9	<0.001
Overall bilateral lobe thickness (mm)	7.1 (5.6, 8.9)	6.3 (5.1, 7.5)	<0.001
CT attenuation (HU)	-61.4 (-80.1, -23.0)	-59.8 (-82.5, -39.7)	0.188

AP, anteroposterior; BCLC, Barcelona Clinic Liver Cancer; CT, computed tomography; HBV, hepatitis B virus; HCC, hepatocellular carcinoma; HCV, hepatitis C virus.

### Thymic imaging parameters in female cirrhotic patients with and without HCC

In the female cohort, no significant differences were observed between groups in the distribution of age (*P* = 0.958), hepatitis virus infection status (*P* = 0.850), and Child-Pugh score (*P* = 0.495; **Table 3**). Importantly, there was no significant difference in the parameter values of thymic CT imaging between female patients in the cirrhosis-without-HCC group and those in the cirrhosis-with-HCC group (all *P* > 0.05).

**Table 3:** Baseline clinical characteristics and thymic imaging parameters in female cirrhotic patients with and without HCC.

Variable	Cirrhosis-without-HCC group (n=35)	Cirrhosis-with-HCC group (n=32)	*P*
Age (year)			0.958
≥60	14 (40.0%)	13 (40.6%)	
<60	21 (60.0%)	19 (59.4%)	
Hepatitis virus infection			0.850
HBV-only	29 (82.9%)	28 (87.5%)	
HCV-only	6 (17.1%)	4 (12.5%)	
HBV + HCV	0 (0.0%)	0 (0.0%)	
Child-Pugh score			0.495
5	26 (74.3%)	26 (81.2%)	
6	9 (25.7%)	6 (18.8%)	
BCLC stage			-
0-A	–	20 (62.6%)	
B	–	6 (18.7%)	
C	–	6 (18.7%)	
AP diameter (mm)	13.3 ± 4.5	11.5 ± 4.8	0.116
Transverse diameter (mm)	22.0 ± 7.1	26.0 ± 9.7	0.067
Left lobe length (mm)	16.6 (13.8, 20.8)	15.1 (12.7, 21.7)	0.624
Right lobe length (mm)	15.2 (12.0, 17.7)	16.2 (12.2, 21.2)	0.340
Overall bilateral lobe length (mm)	15.8 (12.9, 18.9)	15.6 (12.7, 21.6)	0.752
Left lobe thickness (mm)	6.2 (5.0, 6.9)	5.7 (5.1, 6.7)	0.400
Right lobe thickness (mm)	6.5 ± 1.6	6.1 ± 1.8	0.301
Overall bilateral lobe thickness (mm)	6.5 ± 1.8	6.0 ± 1.8	0.177
CT attenuation (HU)	-55.4 ± 36.4	-55.1 ± 31.5	0.971

AP, anteroposterior; BCLC, Barcelona Clinic Liver Cancer; CT, computed tomography; HBV, hepatitis B virus; HCC, hepatocellular carcinoma; HCV, hepatitis C virus.

### Thymic imaging parameters in cirrhotic patients with or without HCC after PSM

**Figure 3:**
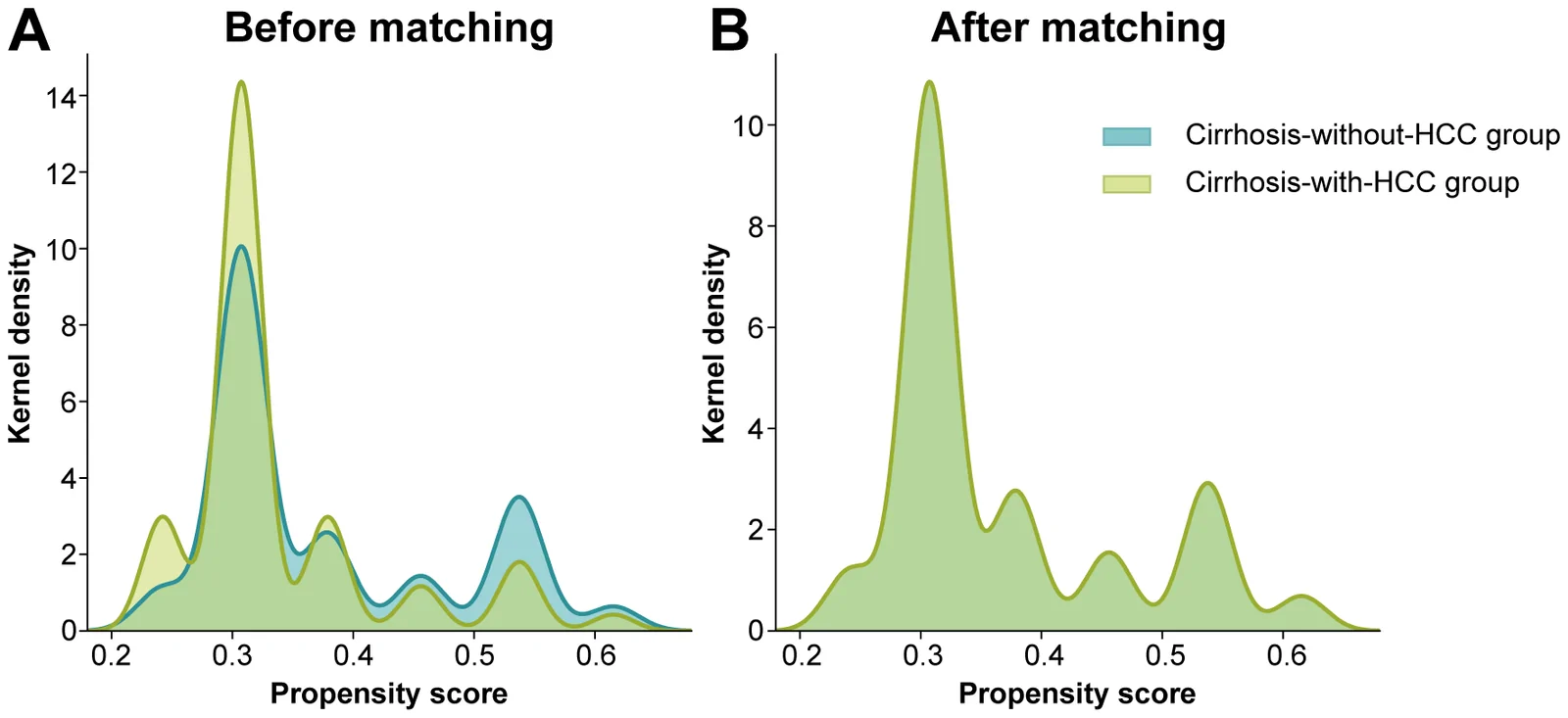
Kernel density plots of propensity scores in the cirrhosis-without-HCC group and the cirrhosis-with-HCC group before matching **(A)** and after matching **(B)**.

To balance baseline characteristics, the sex, age, hepatitis virus infection status, and Child-Pugh score of cirrhotic patients with and without HCC were subjected to PSM at 1:1 ratio. This yielded 115 matched pairs from the original cohort of 121 patients in the cirrhosis-without-HCC group and 223 patients in the cirrhosis-with-HCC group. After matching, the absolute SMD for all four covariates decreased to < 0.001 (**Table 4**). Consistently, the distribution of propensity scores for these two groups, which were clearly divergent before matching (**Figure 3A**), almost completely overlapped after matching (**Figure 3B**), confirming an excellent balance between these two groups.

**Table 4:** Absolute standardized mean differences in baseline characteristics between the cirrhosis-without-HCC and cirrhosis-with-HCC groups before and after PSM.

Variable	|SMD|	
	Before PSM	After PSM
Age	0.032	<0.001
Sex	0.320	<0.001
Hepatitis virus infection	0.048	<0.001
Child-Pugh score	0.120	<0.001

PSM, propensity score matching; SMD, standardized mean difference. An SMD below 0.100 was considered indicative of satisfactory balance, whereas SMDs of 0.100–0.200 and above 0.200 were regarded as reflecting mild and marked imbalance, respectively.

After PSM, all baseline characteristics, including sex, age, hepatitis virus infection status, and Child-Pugh score, were well balanced between the two groups (**Table 5**). Compared with the cirrhosis-without-HCC group, there were significantly smaller thymic dimensions in the cirrhosis-with-HCC group, including anteroposterior diameter (*P* = 0.001), transverse diameter (*P* = 0.027), left lobe length (*P* = 0.002), right lobe length (*P* = 0.002), overall bilateral lobe length (*P* < 0.001), left lobe thickness (*P* < 0.001), right lobe thickness (*P* = 0.004), and overall bilateral lobe thickness (*P* < 0.001). However, there was no significant difference in CT attenuation observed between the cirrhosis-with-HCC and cirrhosis-without-HCC groups (*P* = 0.775).

**Table 5:** Baseline clinical characteristics and thymic imaging parameters in cirrhotic patients with and without HCC after PSM.

Variable	Cirrhosis-without-HCC group (n=115)	Cirrhosis-with-HCC group (n=115)	*P*
Sex			1.000
Male	86 (74.8%)	86 (74.8%)	
Female	29 (25.2%)	29 (25.2%)	
Age (year)			1.000
≥60	22 (19.1%)	22 (19.1%)	
<60	93 (80.9%)	93 (80.9%)	
Hepatitis virus infection			1.000
HBV-only	100 (87.0%)	100 (87.0%)	
HCV-only	15 (13.0%)	15 (13.0%)	
HBV + HCV	0 (0.0%)	0 (0.0%)	
Child-Pugh score			1.000
5	89 (77.4%)	89 (77.4%)	
6	26 (22.6%)	26 (22.6%)	
BCLC stage			-
0-A	–	57 (49.6%)	
B	–	27 (23.5%)	
C	–	31 (26.9%)	
AP diameter (mm)	13.5 (10.2, 16.9)	11.5 (9.0, 14.8)	0.001
Transverse diameter (mm)	22.8 (17.1, 28.3)	20.4 (13.9, 27.4)	0.027
Left lobe length (mm)	17.7 (14.4, 21.2)	14.9 (11.9, 20.0)	0.002
Right lobe length (mm)	17.7 (14.7, 21.6)	15.3 (12.4, 19.3)	0.002
Overall bilateral lobe length (mm)	17.7 (14.5, 21.2)	15.2 (12.2, 19.5)	<0.001
Left lobe thickness (mm)	6.7 (5.8, 8.7)	6.0 (5.0, 6.8)	<0.001
Right lobe thickness (mm)	7.1 (5.6, 8.4)	6.2 (4.8, 7.5)	0.004
Overall bilateral lobe thickness (mm)	6.8 (5.6, 8.5)	6.1 (5.0, 7.4)	<0.001
CT attenuation (HU)	-57.3 (-79.7, -25.0)	-58.3 (-75.9, -36.9)	0.775

AP, anteroposterior; BCLC, Barcelona Clinic Liver Cancer; CT, computed tomography; HBV, hepatitis B virus; HCC, hepatocellular carcinoma; HCV, hepatitis C virus; PSM, propensity score matching.

### Thymic imaging parameters in patients with HCC at BCLC stages 0–A or B–C

Among the 223 patients with cirrhosis-related HCC, 116 were classified as BCLC stage 0–A and 107 as BCLC stage B–C. Before PSM, their baseline characteristics were generally comparable between the two groups, except for the Child-Pugh score, with a higher proportion of patients with a Child-Pugh score of 5 in the BCLC stage 0–A group than in the BCLC stage B–C group (87.1% vs. 74.8%, *P* = 0.019; **Table 6**). However, there was no significant difference in the parameters of thymic CT imaging between the BCLC 0–A and B–C groups, including anteroposterior diameter, transverse diameter, left, right, and overall bilateral lobe lengths, left, right, and overall bilateral lobe thicknesses, and CT attenuation (all *P* > 0.05; **Table 6**).

**Table 6:** Baseline clinical characteristics and thymic imaging parameters in patients with cirrhosis-related HCC at BCLC stages 0-A versus B-C before PSM.

Variable	HCC at BCLC stages 0-A (n=116)	HCC at BCLC stages B-C (n=107)	*P*
Sex			0.200
Male	96 (82.8%)	95 (88.8%)	
Female	20 (17.2%)	12 (11.2%)	
Age (year)			0.416
≥60	28 (24.1%)	21 (19.6%)	
<60	88 (75.9%)	86 (80.4%)	
Hepatitis virus infection			0.290
HBV-only	94 (81.0%)	94 (87.9%)	
HCV-only	21 (18.1%)	12 (11.2%)	
HBV + HCV	1 (0.9%)	1 (0.9%)	
Child-Pugh score			0.019
5	101 (87.1%)	80 (74.8%)	
6	15 (12.9%)	27 (25.2%)	
AP diameter (mm)	12.0 (9.3, 15.4)	12.2 (9.1, 15.3)	0.973
Transverse diameter (mm)	20.5 (13.9, 28.4)	21.2 (15.6, 28.2)	0.452
Left lobe length (mm)	15.6 (12.4, 21.4)	16.6 (13.3, 20.2)	0.973
Right lobe length (mm)	16.1 (12.2, 20.2)	16.4 (13.8, 19.5)	0.492
Overall bilateral lobe length (mm)	15.8 (12.3, 20.7)	16.4 (13.5, 19.9)	0.571
Left lobe thickness (mm)	5.8 (4.8, 7.3)	6.3 (5.2, 7.4)	0.120
Right lobe thickness (mm)	6.3 ± 1.9	6.5 ± 1.9	0.540
Overall bilateral lobe thickness (mm)	6.0 (5.0, 7.4)	6.4 (5.2, 7.6)	0.101
CT attenuation (HU)	-59.6 ± 31.7	-56.7 ± 30.8	0.493

AP, anteroposterior; BCLC, Barcelona Clinic Liver Cancer; CT, computed tomography; HBV, hepatitis B virus; HCC, hepatocellular carcinoma; HCV, hepatitis C virus; PSM, propensity score matching.

To further adjust for baseline imbalance, the sex, age, hepatitis virus infection status, and Child-Pugh score of those HCC patients were subjected to PSM at 1:1 ratio, yielding 83 matched pairs. After matching, the absolute SMDs for all four covariates decreased to < 0.001 (**Table 7**), and all baseline characteristics were well balanced between the two groups (**Table 8**). Consistently, there was no significant difference in any parameter of thymic CT imaging after PSM, including AP diameter (*P* = 0.843), transverse diameter (*P* = 0.172), overall bilateral lobe length (*P* = 0.535), overall bilateral lobe thickness (*P* = 0.144), and CT attenuation (*P* = 0.438; **Table 8**).

**Table 7:** Absolute standardized mean differences in baseline characteristics between patients with cirrhosis-related HCC at BCLC stages 0–A and B–C before and after PSM.

Variable	|SMD|	
	Before	After
Age	0.113	<0.001
Sex	0.190	<0.001
Hepatitis virus infection	0.184	<0.001
Child-Pugh score	0.282	<0.001

PSM, propensity score matching; SMD, standardized mean difference. An SMD below 0.100 was considered indicative of satisfactory balance, whereas SMDs of 0.100–0.200 and above 0.200 were regarded as reflecting mild and marked imbalance, respectively.

**Table 8:** Baseline clinical characteristics and thymic imaging parameters in patients with cirrhosis-related HCC at BCLC stages 0-A versus B-C after PSM.

Variable	HCC at BCLC stages 0-A group (n=83)	HCC at BCLC stages B-C group (n=83)	*P*
Sex			1.000
Male	77 (92.8%)	77 (92.8%)	
Female	6 (7.2%)	6 (7.2%)	
Age (year)			1.000
≥60	15 (18.1%)	15 (18.1%)	
<60	68 (81.9%)	68 (81.9%)	
Child-Pugh score			1.000
5	70 (84.3%)	70 (84.3%)	
6	13 (15.7%)	13 (15.7%)	
Hepatitis virus infection			1.000
HBV-only	74 (89.2%)	74 (89.2%)	
HCV-only	9 (10.8%)	9 (10.8%)	
HBV + HCV	0 (0.0%)	0 (0.0%)	
AP diameter (mm)	12.1 ± 4.3	12.3 ± 4.2	0.843
Transverse diameter (mm)	19.0 (13.6, 27.0)	21.2 (15.8, 28.2)	0.172
Left lobe length (mm)	16.3 ± 5.5	16.3 ± 4.9	0.983
Right lobe length (mm)	15.9 ± 5.2	16.6 ± 4.2	0.371
Overall bilateral lobe length (mm)	16.1 ± 5.3	16.4 ± 4.6	0.535
Left lobe thickness (mm)	6.0 ± 1.8	6.3 ± 1.6	0.199
Right lobe thickness (mm)	6.2 ± 1.8	6.5 ± 1.8	0.426
Overall bilateral lobe thickness (mm)	6.1 ± 1.8	6.4 ± 1.7	0.144
CT attenuation (HU)	-58.6 ± 32.6	-54.9 ± 29.7	0.438

AP, anteroposterior; BCLC, Barcelona Clinic Liver Cancer; CT, computed tomography; HBV, hepatitis B virus; HCC, hepatocellular carcinoma; HCV, hepatitis C virus; PSM, propensity score matching.

## Discussion

Thymic involution has been implicated in tumorigenesis, malignant progression, and antitumor immunity (71, 72). However, whether HCC occurrence is associated with structural thymic involution in humans, particularly among patients with liver cirrhosis, remains unclear. Therefore, this retrospective study investigated the association between HCC occurrence and the parameters of thymus CT imaging in patients with compensated viral cirrhosis.

To the best of our knowledge, this study is the first to show that, among patients with compensated viral cirrhosis, those with HCC have significantly reduced CT-derived thymic dimensions compared with those without HCC. This difference mainly centered on reductions in the anteroposterior diameter, lobe length, and lobe thickness, and was further evidenced after PSM (68, 73–76), which balanced sex, age, hepatitis virus infection status, and Child-Pugh score between these two groups. These findings suggest that HCC occurrence is associated with structural thymic involution under a shared cirrhotic background. In general, radiographic thymic morphology is closely associated with functional thymic activity, and CT provides a widely used non-invasive approach for assessing structural changes in the thymus. A larger thymic volume generally corresponds to greater thymic output. For example, in patients with HIV infection, larger thymic volumes have been significantly associated with higher frequencies of CD4^+^ T-cell receptor excision circles (TRECs), stable by-products of T-cell receptor rearrangement that are commonly used as surrogate markers of recent thymic emigrants and thymic output (77). Moreover, CT-estimated thymic tissue volume has been shown to decline exponentially with age, paralleling the well-established age-related decline in thymic function (78). However, thymic function was not directly assessed in the present study. Therefore, the reduced thymic dimensions observed in our study should be interpreted primarily as radiographic indicators of altered thymic integrity that may be compatible with accelerated immune aging, rather than as definitive evidence of impaired thymopoiesis. Future studies integrating thymic imaging with functional immune assessments, such as TREC quantification and phenotypic characterization of recent thymic emigrants, are warranted to further clarify the immunological significance of HCC-associated thymic involution.

Previous experimental studies have provided evidence supporting a potential role of HCC in accelerating immune aging. In a mouse model of H22 HCC, Sun et al. (36) reported that HCC development induced thymic atrophy that was characterized by reduced thymus weight and thymic index, disruption of the thymic epithelial network, and increased extracellular matrix deposition. Although they did not detect an increase in the frequency of apoptotic thymocytes, they did observe an increased proportion of mature single-positive CD4^+^ and CD8^+^ thymocytes, together with reduced frequency of peripheral T-cells, and upregulated intrathymic Tα1 and Wnt4, leading them to propose that ineffective peripheral antitumor immunity may drive compensatory thymocyte differentiation and maturation, ultimately contributing to thymic cellular depletion and atrophy. In an orthotopic HCC cachexia model, Huang et al. (79) further showed that HCC cachexia remodeled the thymic microenvironment by inducing a maturation disorder of medullary fibroblasts (an increase in immature and a decrease in mature medullary fibroblasts), reducing the expression of LtβR, Mmp9, and Ccl19, as well as tissue-restricted antigen (TRA)-related genes, and impairing T-cell negative selection in mice (79). Upstream of these changes, cancer-cachexia-associated CD45^+^ erythroid progenitor cells induced CD34^+^ progenitor-cell death and thereby reduced functional medullary fibroblasts. Together, these studies suggest that HCC may accelerate thymic involution through abnormal antitumor immune feedback and cachexia-associated thymic microenvironment remodeling. Nevertheless, whether thymic involution in humans acts as either a consequence of HCC or a pre-existing host factor that facilitates HCC development, or both, requires prospective longitudinal investigations.

Sex-stratified analysis showed that, among male patients, most CT-derived thymic dimensions were significantly smaller in the cirrhosis-with-HCC group compared with the cirrhosis-without-HCC group. By contrast, there was no statistically significant difference in CT-derived thymic parameters among female patients. These findings suggest that the association between HCC occurrence and reduced thymic dimensions may be more pronounced in male patients. One possible explanation may involve androgen-related thymic involution, although this hypothesis remains speculative and requires further validation. Men are chronically exposed to higher levels of circulating testosterone than women, and previous studies have shown that testosterone can promote glucocorticoid synthesis by thymocytes, thereby activating the glucocorticoid receptor signaling in an autocrine manner to induce thymocyte apoptosis (80, 81). In addition, testosterone can act through androgen receptors in thymic epithelial cells to downregulate Dll4 expression, impair T-cell lineage commitment of thymic progenitors to reduce thymocyte generation (82, 83). Beyond its effects on the thymus, androgen signaling has also been implicated in both the male predominance and progression of HCC (84–86). Therefore, the androgen-related pathways may represent a potential shared mechanism linking thymic involution and hepatocarcinogenesis in male patients, which may partly explain the more pronounced reduction in thymic dimensions observed in male patients with HCC in the present study. However, the absence of significant differences in the female subgroup should be interpreted cautiously because the number of female patients was relatively small, which may have limited the statistical power of this subgroup analysis.

There was no statistically significant difference in thymic CT attenuation in any analysis, including the overall cohort before and after PSM, as well as the male and female subgroups. Several factors may explain this finding. First, thymic dimensions and CT attenuation represent two distinct aspects of thymic involution: thymic dimensions reflect the residual size of the thymic compartment, whereas CT attenuation mainly reflects the degree of fatty replacement within thymic tissue (61). More importantly, thymic fatty replacement progresses continuously with age (87, 88). CT attenuation values decline steadily after puberty, and thymic tissue is largely replaced by adipose tissue in many adults by the late 50s (89). In the present study, the two groups had comparable age distributions and consisted mainly of adult patients, in whom thymic fatty replacement may already have progressed substantially, leaving CT attenuation values within a similarly low range in both groups.

The absence of significant difference between BCLC stage 0–A and B–C suggests that thymic involution may be more closely related to the presence of HCC than to BCLC stage progression. One possibility is that thymic changes emerge early during hepatocarcinogenesis and do not necessarily worsen in parallel with conventional tumor stage. Nevertheless, this interpretation should not be overextended, because BCLC stage is a composite clinical staging system that integrates tumor burden, liver function, and performance status, but is not specifically designed to reflect tumor-associated immune dysregulation, systemic inflammation, or thymic stress. In addition, the limited sample size and the combined analysis of BCLC stages B and C may have obscured biologically meaningful heterogeneity. Future studies with larger cohorts should perform more granular analyses according to tumor burden, microvascular invasion, AFP level, systemic inflammatory indices, cachexia or sarcopenia status, and immunotherapy outcomes as well as peripheral T cell immunity to clarify whether thymic involution is linked to specific tumor-immune or host inflammatory phenotypes rather than to BCLC stage itself.

From a clinical perspective, the association between reduced CT-derived thymic parameters and HCC occurrence may have potential translational implications. Because thymic dimensions can be readily assessed on routine chest CT, they may represent accessible imaging biomarkers that warrant further evaluation for identifying patients with viral cirrhosis at increased risk of developing HCC. Beyond HCC risk assessment, CT-based thymic assessment may also have potential value for prognostic evaluation and risk stratification among patients with established HCC, although its prognostic performance and incremental clinical value remain to be determined. In the era of cancer immunotherapy, CT-derived thymic parameters may additionally provide a non-invasive surrogate of host immune status and could therefore be explored as candidate imaging biomarkers for predicting responses to immune checkpoint inhibitors or for identifying patients who might benefit from immune-modulating strategies, such as thymosin alpha-1 (90). However, these potential applications remain hypothesis-generating and require validation in prospective longitudinal studies incorporating functional immune biomarkers, treatment-response data, and established clinical prognostic factors.

The results of the current study should be interpreted in the context of several limitations. First, the retrospective and single-center design may limit the generalizability and reproducibility of our findings. Furthermore, our cohort was restricted to patients with Child-Pugh class A, virus-related (predominantly HBV) cirrhosis, and patients with HCC were further limited to those who underwent radical resection. Consequently, our findings may not be generalizable to HCC arising from non-viral etiologies (such as alcohol-associated or MASLD), to patients with decompensated cirrhosis, or to those with unresectable disease. Whether HCC-associated thymic involution occurs across these broader populations warrants further investigation. Second, the cross-sectional design precludes determination of the causal direction between thymic involution and HCC occurrence. Whether thymic involution precedes and facilitates HCC development through impaired immune surveillance, whether HCC accelerates thymic involution, or whether both processes coexist cannot be established from the current data. Third, although PSM balanced age, sex, hepatitis virus infection status, and Child-Pugh score, residual confounding by BMI, smoking status, metabolic comorbidities, nutritional status, sarcopenia or cachexia, systemic inflammation, and antiviral treatment could not be fully excluded. Fourth, thymic assessment was based on two-dimensional CT-derived linear measurements rather than volumetric segmentation or radiomics-based assessment, and CT attenuation may have been affected by differences in CT acquisition protocols. Finally, functional markers of thymic output and immune aging, such as signal-joint TREC levels, naïve T-cell proportions, and T-cell receptor repertoire diversity, were unavailable.

## Conclusion

In conclusion, this study provides the first clinical evidence that HCC occurrence is associated with reduced CT-derived thymic dimensions in patients with compensated viral cirrhosis. These findings introduce a novel perspective on HCC immunobiology by extending consideration beyond the local tumor microenvironment to systemic host immune competence. The association remained evident after PSM for age, sex, hepatitis virus infection status, and Child-Pugh score and was particularly pronounced in male patients. Prospective longitudinal studies integrating thymic imaging with functional immune biomarkers are warranted to clarify the causal direction and clinical significance of HCC-associated thymic involution.

## Data Availability

The original contributions presented in the study are included in the article/supplementary material. Further inquiries can be directed to the corresponding authors.
